# A semi-synchronous label propagation algorithm with constraints for community detection in complex networks

**DOI:** 10.1038/srep45836

**Published:** 2017-04-04

**Authors:** Jia Hou Chin, Kuru Ratnavelu

**Affiliations:** 1 Institute of Mathematical Science, University of Malaya, Kuala Lumpur, Malaysia

## Abstract

Community structure is an important feature of a complex network, where detection of the community structure can shed some light on the properties of such a complex network. Amongst the proposed community detection methods, the label propagation algorithm (LPA) emerges as an effective detection method due to its time efficiency. Despite this advantage in computational time, the performance of LPA is affected by randomness in the algorithm. A modified LPA, called CLPA-GNR, was proposed recently and it succeeded in handling the randomness issues in the LPA. However, it did not remove the tendency for trivial detection in networks with a weak community structure. In this paper, an improved CLPA-GNR is therefore proposed. In the new algorithm, the unassigned and assigned nodes are updated synchronously while the assigned nodes are updated asynchronously. A similarity score, based on the Sørensen-Dice index, is implemented to detect the initial communities and for breaking ties during the propagation process. Constraints are utilised during the label propagation and community merging processes. The performance of the proposed algorithm is evaluated on various benchmark and real-world networks. We find that it is able to avoid trivial detection while showing substantial improvement in the quality of detection.

Over the decade, network analysis has been widely applied in various research fields such as biology, transportation, sociology and bibliometric studies^1^,^2^,^3^,^4^. Complex networks posses features that provide insight into these properties, with a majority of the real-world complex networks consisting of a network feature called the community structure. A community in a complex network is defined as a set of nodes that are densely connected to each other in a group, while they are loosely connected with the rest of the network^5^. Naturally, nodes with similar attributes will be more likely to form a community. Thus, in principal, one can acquire the functions, traits or properties of a group of individuals by investigating a community. Given the practicality of studying the community structure in complex networks, community detection emerges as a popular research topic. Consequently, a large number of community detection algorithms have been developed for the purpose of uncovering the community structure in complex networks^6^.

The label propagation algorithm (LPA)^7^ was first introduced in 2007, as a community detection algorithm that requires less computational time. The objective of the LPA is to allocate each node into a community with the most number of its neighbouring nodes. The simplicity and near linear complexity of the LPA makes it feasible to detect communities in huge networks with millions of nodes. However, there are some pronounced issues in the LPA that affect its performance. The randomness that is induced in its update sequences and tie breaking processes cause the LPA to return multiple detections, thus making it a non-deterministic detection algorithm. Furthermore, in networks with a weak community structure, the LPA is unable to detect any meaningful community. As a consequence, the LPA detects only one community (trivial detection) in such networks.

The relative simplicity of LPA, coupled with these issues, led scientists to seek improvements and enhancements in this algorithm. Leung *et al*.^8^ introduced link preferential and hop attenuation to handle the tie breaking cases. Modularity was implemented into the LPA by Barber and Clark^9^, while Liu and Murata^10^ further improved it by merging the detected communities to further increase the modularity. Xie *et al*.^11^ proposed a modified LPA, called the speaker-listener based LPA (SLPA), that can detect overlapping communities. Aside from their SLPA, Xie and Szymanski^12^ also developed LabelRank, a LPA variant that implemented a Marcov Cluster Algorithm in order to stabilise LPA. They further improved the LabelRank with an update rule, hence speeding up the LPA^13^. A modified LPA that utilises a prediction of the percolation transition was proposed by Zhang *et al*.^14^. That algorithm is able to delay the formation of monster size communities, which reduces the chance of trivial detection. The NIBLPA^15^ is a node-influence based LPA that tackles the randomness issue in the LPA. The influence scores, of the nodes in a network, are used to determine the update sequences as well as breaking ties between multiple communities during the propagation processes. The most recent LPA variants include the SpeakEasy^16^, LINSIA^17^ and CLPA-GNR^18^ algorithms. SpeakEasy is a LPA variant that specialises in the detection of overlapping communities in biological networks. It employs both the neighbouring and global information of a network so that the combination of that information can yield accurate detection. The ability of LINSIA to control the propagation processes allows it to reveal hub and outlier nodes, apart from detecting overlapping communities in a network. CLPA-GNR is a modified LPA that implements constraints at different stages of the algorithm, while updating the solo and grouped nodes separately. Furthermore, it can obtain deterministic detections in undirected and unweighted networks by removing the randomness in the LPA. Even though much effort has already been expended in getting rid of the disadvantages and in enhancing the advantages of the LPA, the improvement of the LPA in terms of robustness and stability remains an open question.

## Semi Synchronous Constrained Label Propagation Algorithm (SSCLPA)

The implementation of constraints and fixed update sequences in the CLPA-GNR allow it to produce accurate and deterministic detection. However, the drawback of the CLPA-GNR is its tendency of obtaining trivial detection in networks with weak community structure^18^. Hence, in this work, we address this issue and propose a new LPA variant called SSCLPA, which is an improved CLPA-GNR. The proposed algorithm can detect disjoint communities in undirected and unweighted networks. It draws on the essence of the CLPA-GNR such as the constraints and fixed update sequence, and further enhances them. As a result, the SSCLPA is able to avoid trivial detection and still be able to obtain deterministic and accurate detection.

Similar to its predecessor, the proposed algorithm consists of constraints that are applied at various stages of the algorithm. The restrictions are gradually relaxed towards the end of the algorithm. A new constraint is implemented in the SSCLPA, where communities that reach certain threshold of strength values are exempted from the propagation or merging processes. This new form of constraint is crucial in delaying the formation of monster size communities, hence allowing the growth of other communities. By limiting the growth of specific communities, the chances of getting trivial detection can be eliminated. Instead of the mutual neighbour score (*MNS*) that is used in the CLPA-GNR, the Sørensen-Dice index (*SDI*) is implemented as the similarity score in SSCLPA. Similar to the function of the *MNS* in the CLPA-GNR, *SDI* is used in the early stage of the algorithm to detect initial communities. Aside from that, it can substitute the capacity score in the CLPA-GNR to break ties between multiple communities during the propagation processes.

In both the CLPA-GNR and SSCLPA, nodes are categorised into two types, namely the solo and grouped nodes. But, unlike in the CLPA-GNR where all the propagation processes are asynchronous, the solo nodes undergo synchronous updates while grouped nodes are subjected to asynchronous updates in the SSCLPA. The synchronous updates of solo nodes can speed up the propagation process without sacrificing the accuracy of the detection. There are also difference in the rules for the update sequence in CLPA-GNR and SSCLPA. In the CLPA-GNR, the degree of the nodes is the only criterion in deciding the update sequences. However, in the SSCLPA, the number of neighbouring nodes that are also solo nodes is also taken into account.

In general, the SSCLPA will do an initial detection by using similarity score, which creates large amount of small communities. The propagation process involves the allocation of nodes into detected communities, while the merging process attempts to reduce the number of communities by merging them. These processes are repeated throughout the algorithm until convergence in the labels is achieved. The details of the SSCLPA are explained in the Method section.

## Results

The SSCLPA is tested on various benchmark networks before it is implemented on any real-world network. Three types of benchmark networks are employ in this study, namely the Lancichinetti-Fortunato-Radicchi (LFR)^19^,^20^, Girvan-Newman (GN)^5^, and Relaxed Caveman (RC)^21^,^22^ benchmark networks. It must be noted that at this time both the benchmark and real-world networks are undirected and unweighted with disjoint communities. The evaluation criterion for networks with ground truth communities is the normalised mutual information (*NMI*)^23^. The value of the *NMI* ranges from 0 to 1, where *NMI* = 1 when two partitions are identical. If a partition is totally independent of another partition, then *NMI* = 0. On the other hand, the modularity (*Q*)^24^ and modularity density (*Q*_*ds*_)^25^ are used to evaluate the quality of detection in networks without the ground truth communities. A good detection yields high values of *Q* and *Q*_*ds*_.

The performance of the proposed algorithm is also compared to the other community detection methods: LPA^7^, CLPA-GNR^18^, GANXiS (or SLPA)^11^,^26^,^27^, the Ronhovde and Nussinov algorithm (RN)^28^, Blondel^29^, and Infomap^30^. LPA is the original label propagation algorithm, while the CLPA-GNR and GANXiS are the aforementioned LPA variants. In addition, the RN is a spin-glass type Potts model community detection algorithm. This algorithm is not only good in detecting heterogeneous sized communities in a network, but it is also free from resolution-limit. The Blondel algorithm is an effective modularity optimisation detection method, that can detect communities heuristically in a relatively short computational time. Lastly, Infomap detects communities by optimising the map equation while minimising the description length of a random walker. As GANXiS, LPA and Blondel do not produce a deterministic detection, they are executed 100 times for each network and the detection that yields the highest *Q* value is chosen for the purpose of comparison.

### Lancichinetti-Fortunato-Radicchi benchmark (LFR)

The LFR benchmark networks are the most popular benchmark networks for community detection algorithms, as they contain features that are common in the real-world networks. Furthermore, the degree of nodes and the sizes of the communities in the generated LFR synthetic networks are always heterogeneous. One of the most important parameters in the LFR networks is the mixing parameter, *μ*, which represents the average percentage of edges that connect a pair of nodes from different communities. Note that the strength of the community structure decreases as the value of *μ* increases.

We generated 4 groups of LFR networks in this investigation. The average and maximum degrees are fixed at *k*_*avg*_ = 20 and *k*_*max*_ = 50. On the other hand, the exponent for the degree sequence and the exponent for the community size distribution are fixed at *γ* = 2 and *β* = 1. The rest of the parameters are depicted in Table 1.

**Figure 1:**
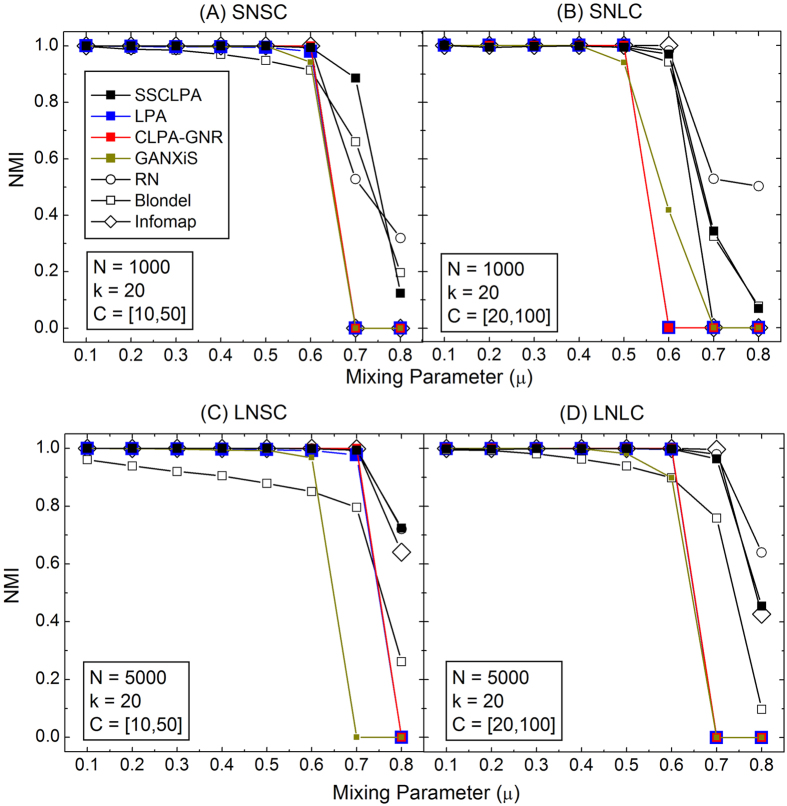
The *NMI* comparison on the undirected and unweighted LFR benchmark networks, with various network sizes and community sizes. The notations *N, k* and *C* refer to the size of networks, average degree and the size of communities, respectively. See also the caption on the plots.

Figure 1 shows the results for the various detection algorithms on the 4 groups of LFR networks. Algorithms that yield *NMI* = 0 indicate that those algorithms can only detect a single community in the networks. Thus, the detection is trivial and the algorithms cannot uncover any community structure in those networks. The present algorithm manages to detect community structure in all the groups of the LFR networks, even in networks with high *μ* values. The other LPA based algorithms, including the original LPA, get trivial detection when *μ* ≥ 0.6. Infomap also faces a similar problem at high *μ* values. We highlight that not only does the SSCLPA detect communities at high *μ* values (*μ* ≥ 0.7), the quality of the detection is also good. For instance, in the SNSC case (Fig. 1(A)), the *NMI* of the present algorithm is significantly higher than the other algorithms at *μ* = 0.7. The *NMI* values of the SSCLPA in the SNLC, LNSC and LNLC cases (Fig. 1(B–D)) are close to 1, which indicates near perfect detection. Although the performance of the present algorithm is not as good as RN at *μ* = 0.8 in most of the networks, the quality of its detections is still highly acceptable. One of the reasons for the discrepancies between the SSCLPA and RN algorithms is the number of detected communities in networks with *μ* = 0.8. The number of detected communities in those networks by using the present algorithm is always smaller than the number of ground truth communities. On the contrary, the RN yields a large number of small size communities in those networks. Nonetheless, the overall performance of SSCLPA is good and it outperforms the other LPA based algorithms when applied to the LFR benchmark networks.

### Girvan-Newman benchmark (GN)

There are 128 nodes, which are divided equally into 4 communities with 32 nodes each, in the GN networks. The parameters, *P*_*in*_ and *P*_*out*_, refer to the probabilities of defining an edge as an intra-community or inter-community edge. It is important for 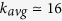 when deciding the values of *P*_*in*_ and *P*_*out*_. GN networks can be generated by using the LFR networks generator, where *μ* is used to represent *P*_*in*_ and *P*_*out*_. The following parameters are employed to generate the GN networks with the LFR networks generator: *N* = 128, *k*_*avg*_ = *k*_*max*_ = 16, *C* = 32, *γ* = *β* = 0, and *μ* = 0–0.8.

**Figure 2:**
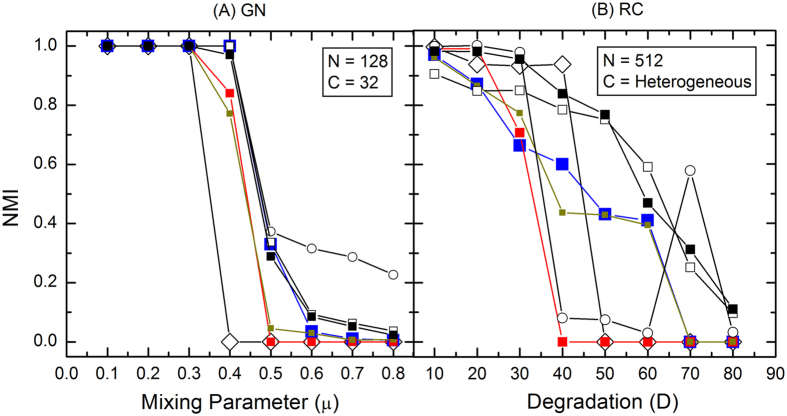
The *NMI* comparison on the undirected and unweighted GN and RC networks. The legend is the same as in Fig. 1.

The comparison between the results of the SSCLPA and the other algorithms in a GN benchmark is depicted in Fig. 2(A). Once again, the SSCLPA yields the best detection across the *μ* values among the LPA based algorithms in this benchmark test. Furthermore, it outperforms Infomap and its performance is on par with Blondel. Although the *NMI* values of the proposed algorithm are again not as high as the RN at *μ* ≥ 0.6, the SSCLPA still manages to detect 4 communities in those GN networks. On the other hand, similar to the LFR cases, RN detects far more communities than the number of ground truth communities in those networks.

### Relaxed Caveman benchmark (RC)

The RC benchmark networks have 512 nodes with 16 communities of highly heterogeneous size. Initially, a RC network consists of 16 isolated k-cliques. Similar to the role of *μ* in the LFR benchmark networks, here a parameter known as the Degradation (*D*)^31^ is implemented to progressively weaken the community structure of the network. As the value of *D* increases, the number of intra-community edges that are converted to inter-community edges is increased. The value of *D* is varied from 10% to 80% in this work. As shown in Fig. 2(B), the performance of the SSCLPA is exceptional in the RC benchmark networks. Generally, it has higher a *NMI* than all the LPA based algorithms. Moreover, instead of getting trivial detection like the other LPA based algorithms at the higher *D* values, the results of the proposed algorithm are comparable to those from Blondel which has the best performance in the RC networks. We note that the RN shows a sudden spike in the *NMI* value at *D* = 70%. Apparently, this phenomenon is caused by the tendency of RN to detect large number of communities in networks with weak community structure. This tendency of RN renders its detection unreliable in finding meaningful communities in those kind of networks.

### Real-World Networks

The real-world networks that are used in this study are summarised in Table 2 and the detection results are depicted in Tables 3, 4 and 5. These networks are often employed in the testing of community detection algorithms. As shown in Table 3, the detection performance of the SSCLPA agrees fairly well with the ground truth communities in the Zachary, Dolphins and Football networks. In fact, it achieves the highest *NMI* value in the Football network and it is also the second best algorithm in the Zachary network. Its performance in the Pol-books network is less than desirable, but this is compensated for by having the second highest *Q*_*ds*_ value (see Table 5).

Undoubtedly, the Blondel algorithm, which focuses on the optimisation of the *Q* value, can obtain the highest *Q* values in almost all of the real-world networks (see Table 4). Nonetheless, the performance of the SSCLPA in terms of the *Q* values is acceptable, considering the fact that its *Q* values are within 6% of the highest *Q* values in networks with sizes lesser than 10000 nodes, except for the Email network, which is ~8% lower than then best *Q* value. It can be observed that the SSCLPA underperforms in term of the *Q* in large networks such as the Pretty Good Privacy, Astro-ph and Brightkite networks. However, the SSCLPA has the second highest *Q*_*ds*_ after the *Q*_*ds*_ of the GANXiS in those networks (see Table 5). Furthermore, it is worth noting that its *Q*_*ds*_ is higher than those of the Infomap and Blondel algorithms. In general, the proposed algorithm outperforms most of the other algorithms except for the GANXiS in term of *Q*_*ds*_. Instead, it can be observed that the SSCLPA has the best detection performance in terms of *Q*_*ds*_ in the Football and Jazz networks.

The computational time of various community detection algorithm is compared in Table 6. Since in general the CLPA-GNR runs slower than the SSCLPA, we do not report the computational time of the CLPA-GNR. As a LPA variant that yields deterministic detection, the SSCLPA runs in reasonable time in large networks. In general, the LPA can run faster than the proposed algorithm, but the time that are depicted in the table is the total time for 100 runs. Thus, it is not guaranteed that the LPA can produce its best detection within those runs. In fact, larger number of runs is needed by LPA as the size of the networks increases. GANXiS, which is also a LPA variant, requires longer computational time despite its good performance in terms of *Q* and *Q*_*ds*_ in those networks. For instance, GANXiS consumes 1741s to produce 10 detection results. Although the Infomap and Blondel algorithms can complete the detection within 10 s, but their low *Q*_*ds*_ values in those networks cannot be overlooked.

## Discussion

The SSCLPA addresses both the randomness and trivial detection issues in the LPA. It inherits the prominent features of our earlier CLPA-GNR and further enhances them. In particular its update sequences are fixed based on the degree and the number of solo neighbouring nodes. Furthermore, the *SDI* is used in early detection and to break ties during the propagation processes. Constraints, such as the conditions of propagating labels and the exemption of the communities, are introduced at various stages of the SSCLPA. These restrictions help the proposed algorithm to avoid trivial detection. The process of dividing nodes into two groups, which are then updated separately, ensures the good quality of the detections. As the random elements are eradicated in the SSCLPA, it is able to provide deterministic detection. The performance of the proposed algorithm in both the benchmark and real-world networks is excellent, regardless of the sizes of the networks. As a LPA variant, the proposed algorithm does not obtain any trivial detection, and it can detect high quality communities in terms of the *NMI, Q* and *Q*_*ds*_ metrics. Moreover, the SSCLPA is a time efficient community detection algorithm that can run in reasonable time, considering that fact that it is able to provide good and deterministic detection. The results in this work show that the SSCLPA is a promising community detection algorithm that works well in detecting disjoint communities in undirected and unweighted networks.

There remains, however, rooms for improvement in the performance of the SSCLPA. Other than the *SDI*, better similarity scores can improve the outcomes of the initial grouping stage and breaking the ties more accurately. It would also be interesting to implement different criteria on the exemption of communities, in order to observe the effects of exempting different communities on the final outcomes of the detections. Since the CLP processes are synchronous, they are readily parallelisable. The extension of the SSCLPA into the directed or weighted networks is highly possible, and it is a top priority in our future work.

## Methods

Let *G* = (*V, E*) be a network where 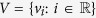 and 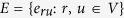 are sets of nodes and edges. In the SSCLPA, nodes are divided into two types, known as the solo and grouped nodes. Solo nodes refer to nodes that are not yet assigned into any community yet, while grouped nodes mean otherwise. Solo and grouped nodes are denoted by 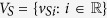 and 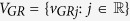, respectively. The labels of *V*_*s*_ and *V*_*GR*_ are updated separately in two propagation processes in the SSCLPA. On the one hand, the size of the detected communities is increased by allocating *V*_*s*_ into the communities. On the other hand, the detected communities are strengthened in term of the density of the communities by reallocating *V*_*GR*_ amongst the detected communities. In the original LPA, all the labels of the nodes are updated in one propagation process. As the labels of the nodes are affected by the labels of the neighbouring nodes during the propagation process, the labels of the nodes are susceptible to the changes of the labels of all the nodes in a network if they are updated in a single propagation process. By separating the updates of the labels of *V*_*S*_ and *V*_*GR*_ in two propagation process, *V*_*S*_ and *V*_*GR*_ are only susceptible to the changes in the labels of *V*_*S*_ and *V*_*GR*_ during their corresponding label propagation processes, respectively. To be precise, the labels of *V*_*S*_ are only affected by the changes in the labels of *V*_*S*_ as the labels of *V*_*GR*_ remain the same throughout the label propagation involving *V*_*S*_. The same concept is applied during the label propagation involving *V*_*GR*_. Hence, this update strategy is a more organised way of updating the labels of nodes than that in the original LPA.

In the proposed algorithm, the labels of *V*_*S*_ are updated synchronously. Thus, an update sequence is not required. However, *V*_*GR*_ is updated asynchronously. The rules for the update sequence in this case are as follows:

1. Nodes are arranged in ascending order based on the number of solo neighbouring nodes. Solo neighbouring nodes are neighbouring nodes that are solo nodes. Nodes with lesser solo neighbouring nodes are updated first.
2. Nodes are then arranged in ascending order based on their degree. Nodes with a lower degree value are updated first.

The update sequence of nodes are decided by update rule 1 first. If multiple number of nodes have the same number of solo neighbouring nodes, then update rule 2 is applied on those nodes. The possible candidate labels of the target nodes are lesser if the number of solo neighbouring nodes is lesser. Furthermore, nodes with a low degree usually serve as the peripheral member nodes in large communities. Hence these update rules prioritise nodes that have a lesser influence on the labels of the other nodes. The rules are implemented in all processes unless otherwise stated.

**Figure 3:**
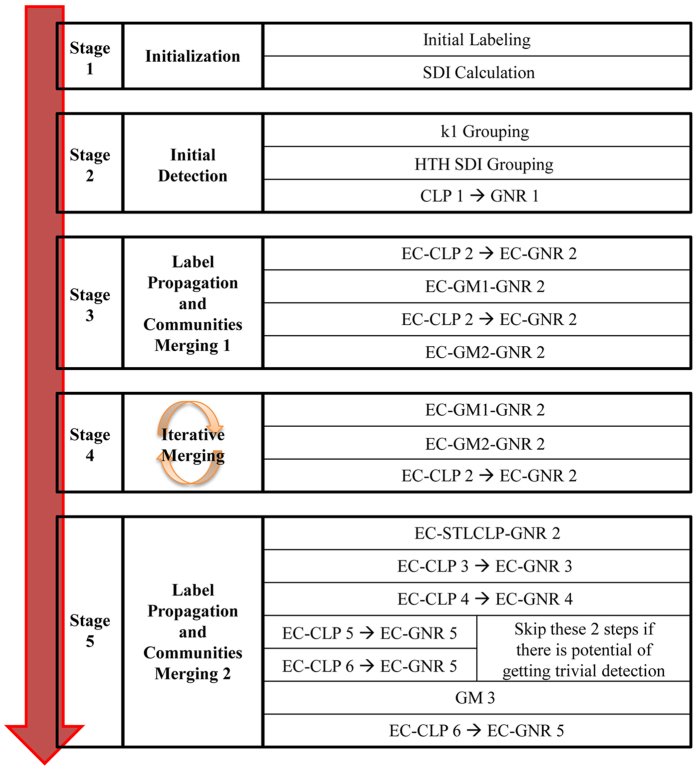
The flowchart of the SSCLPA. In Stage 1, each node is given a unique label. The *SDI* score for each couple of nodes are calculated. In Stage 2, large number of communities are detected by using k1 grouping and HTH *SDI* grouping. The size of detected communities is increased by using CLP 1 and GNR 1 is used to strengthen the communities. CLP 1 → GNR 1 indicates that CLP 1 is executed until there is a convergence in the labels of the nodes before GNR 1 is executed. In Stage 3, the size of communities is further increased by using CLP 2. On the other hand, the number of detected communities are reduced by using GM 1 and GM 2 processes. The dash symbol indicates combination of the processes. For example, EC-CLP 2 shows that EC is executed once before CLP 2 is executed. The number of communities are further reduced in Stage 4. The processes in this stage are executed recursively until it reaches a stability where the number of communities cannot be reduced anymore. There are two ways of proceeding Stage 5. The first way is to execute all the parts in this stage. If trivial detection is obtained at the end of this stage, then one can choose to skip EC-CLP 5 → EC-GNR 5 and EC-CLP 6 → EC-GNR 5 parts. By doing so, SSCLPA can avoid trivial detection. SSCLPA is terminated when it reaches EC-CLP 6 → EC-GNR5 in Stage 5.

There are 4 main processes that are being utilised extensively in the 5 main stages in the proposed algorithm (see Fig. 3). These are now explained in the following subsections.

### Main Processes

#### Exempted Community (EC)

It is common to find that after a few iterations of the LPA, some of the detected communities are far stronger than most of the others. These communities will grow exponentially in the later stages of the LPA. Eventually, this phenomenon may lead to trivial detection, where the LPA only detects a single community that consists of all the nodes in a network. In order to prevent this kind of detection, communities that exceed a strength threshold are exempted from the propagation and merging processes. By doing so, the other communities have the chance to grow without competing with those communities.

Given that 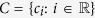 and 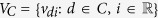 as the set of communities and their corresponding member nodes in the communities, the term 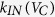 is defined as the intra-community degree. For instance, 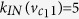 shows that 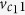 is connected to 5 other member nodes in community 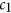. Then, the strength value of the member nodes in the communities is defined as:


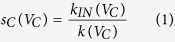


where 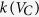 is the degree value of the corresponding member nodes. As consequence, the strength value of the communities can be obtained:


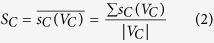


where 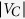 is the number of member nodes in a community.

Finally, 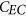 is defined as a set of communities with 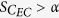. The parameter, *α*, is the strength value that determines the number of communities to be exempted and 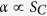. In general, a lower *α* value brings about more exempted communities and we note that this process is executed prior to other processes as *C*_*EC*_ plays a crucial part in those processes.

#### Constrained Label Propagation (CLP)

The CLP is a label propagation process that assigns *V*_*S*_ into detected communities. Nodes are updated synchronously in CLP unless stated otherwise. Let node 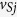 be the targeted node. A community *c*_*i*_ is eligible to claim 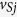 if it fulfils the following conditions:

1. Let 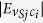 be the total number of edges between 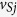 and 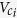. Then 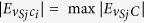.
2. *c*_*i*_ ∉ *C*_*EC*_.
3. If *Q0* is the minimum 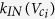 and *Q1* is the first quartile of 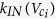, then 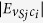 must be larger or equal to *Q0* or *Q1*. This condition is defined differently at various stages of the algorithm.

CLP Condition (1) is simply the label propagation condition of the original LPA. However, this condition does not always increase or retain the strength of the communities after the solo nodes enter the communities. Therefore, CLP Condition (3) is implemented for this purpose. Finally, CLP Condition (2) ensures that the target solo nodes do not enter the exempted communities.

If there is a tie between multiple communities, the mean of the *SDI* (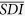) of the competing communities is compared. For example, let there be a tie between communities *c*_1_ and *c*_2_. The targeted node *v*_*S*1_ is connected to *c*_1_ and *c*_2_ via 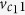, 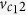 and 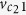, 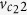, respectively. If the 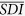 of *v*_*S*1_ with 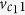, 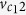 is higher than that for *v*_*S*1_ with 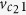, 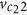, it will enter *c*_1_ provided that the CLP Condition (3) is satisfied. The labels of the nodes remain the same if there is a tie in the 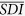.

#### Grouped Nodes Reallocation (GNR)

The function of the GNR is to check the validity of *V*_*GR*_ and reallocate them if necessary. The GNR is identical to the CLP process, but it is implemented on the group nodes *V*_*GR*_ instead of the solo nodes *V*_*S*_. So, similar to the CLP, the GNR needs to fulfil the conditions that are applied on the CLP before *V*_*GR*_ can be reallocated from one community to another. If there is a tie between multiple communities, the mean of the *SDI* (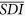) of the competing communities is compared. In contrast to the CLPA, the GNR is an asynchronous label propagation process. As a consequence, *V*_*GR*_ are updated asynchronously. Since the purpose of the GNR is to strengthen the detected communities, it is usually implemented after the CLP or GM processes.

#### Groups Merging (GM)

Start with the largest community in descending order, a couple of communities, *c*_*i*_ and *c*_*j*_, can be merged if the following conditions are met:

1. *c*_*i*_, *c*_*j*_ ∉ *C*_*EC*_.
2. Let 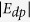 be the number of edges between a couple of communities, where *d, p* ∈ *C* and *d* ≠ *p*. Then, this condition is defined as 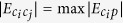.
3. Let 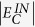 be the total number of intra-community edges in the communities. Two ratios are obtained, where *RatioA* is the average number of edges in *c*_*j*_ and *RatioB* is the average number of edges between *c*_*i*_ and *c*_*j*_. Then this condition is defined as:

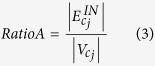



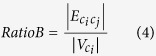



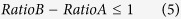


GM Condition (3) is a relaxed version of the merging condition in the CLPA-GNR^18^ where it allows more communities to be merged. Nonetheless, this merging strategy can still maintain the strength of the merged communities to some extent.

### Main Stages

The flowchart of the SSCLPA is depicted in Fig. 3. The details of the stages and their corresponding processes are explained in the following subsections. In general, each stage is a combination of variations in the CLP, GNR and GM processes.

#### Stage 1: Initialisation

In this stage, the nodes are assigned unique initial labels and the *SDI* is calculated.

- Initial labeling: Every single node in a network is given a unique label.
- *SDI* calculation: The *SDI* of all the pairs of nodes, *x* and *y*, in an undirected and unweighted network is calculated:

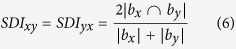


where 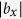 and 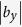 are the number of neighbouring nodes of *x* and *y* respectively. It must be noted that 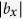 does not include node *y* and vice verse. The term 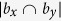 represents the number of mutual neighbouring nodes of *x* and *y*.

#### Stage 2: Initial Detection

This stage aims to detect as many communities as possible in a network. Nodes with one degree are grouped with their sole neighbouring nodes to form communities. Then, more communities are found by using the highest to highest (HTH) *SDI* Grouping. The sizes of the detected communities are increased by using CLP 1 and the communities are strengthen by GNR 1.

- k1 grouping: Nodes with one degree are assigned the label of its sole neighbouring node.
- HTH *SDI* Grouping: Solo nodes *V*_*S*_, in ascending order of degree, are assigned into communities where the member nodes of the communities have the highest *SDI* score with each other. Let 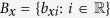 be a set of neighbouring nodes of node *x*, and the highest *SDI* score of node *x* is defined as 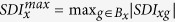. Node *x* will be assigned into a community with a set of nodes, *V*_*M*_ ⊂ *V* if the following conditions are satisfied:
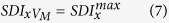

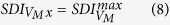
- CLP 1: This is an asynchronous CLP so *V*_*S*_ is updated according to the predefined update sequence. In this CLP, a community *c*_*i*_ must have at least one member node which has the highest *SDI* score with the target solo node 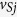, 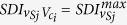. CLP Condition (2) is not required here. CLP Condition (3) is defined as 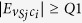. The labels of the nodes remain the same if there is a tie between multiple communities.
- GNR 1: CLP Condition (2) is not required in this GNR. CLP Condition (3) is defined as 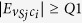 here. The labels of the nodes again remain the same if there is a tie between multiple communities.

#### Stage 3: Label Propagation and Communities Merging 1

In this stage, both the CLP 2 and GNR 2 processes are executed iteratively in order to further increase the sizes of the communities. Every time the CLP 2 is executed, it is followed by the GNR 2 for enhancement purpose. In order to reduce the large number of communities that are detected in Stage 2, GM1 and GM2 are introduced in this stage. Similarly, GNR 2 is executed after the CLP and GM processes in order to strengthen the detected communities.

- CLP 2 & GNR 2: Here we execute the CLP and GNR processes with CLP Condition (3) of 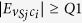.
- GM 1: Execute the GM process on communities with more than 3 member nodes, 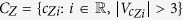. This step prevents the communities with lesser than 4 member nodes from disrupting the merging process, which have the potential of forming monster size communities. The labels of the nodes remain the same if there is a tie between multiple communities.
- GM 2: Unlike GM 1, all the detected communities, regardless of their sizes, can be merged. However, a new condition where the modularity score does not decrease after the merging is added in GM 2. This additional condition controls the merging of the communities with lesser than 4 member nodes. The labels of the nodes again remain the same if there is a tie between multiple communities.

#### Stage 4: Iterative Merging

In Stage 4, GM 1 and GM 2 are executed iteratively to further reduce the number of communities, until the networks reach a stability where the number of communities cannot be further reduced.

#### Stage 5: Label Propagation and Communities Merging 2

In the last stage, STL-CLP is used to boost the size of the communities that do not grow in size during the previous stages. Then, most of the the *V*_*S*_, if not all, will be assigned into communities by using CLPA 3/4/5/6. In order to do so, the constraints on the CLP 3/4/5/6 are gradually relaxed from CLP 3 to 6. Furthermore, the remaining communities will be merged for the last time by using GM 3. As usual, GNR 3/4/5 are used to strengthen the communities after the CLP and GM processes. Similar to the CLP 3/4/5/6, the constraints in the GNR 3/4/5 are gradually relaxed. In networks with a weak community structure, some of the processes are omitted in order to avoid trivial detection. This procedure is explained in the legend of Fig. 3.

- Smallest to largest CLP (STL-CLP): Start from the smallest and proceed to the largest communities, a community *c*_*i*_ will absorb a solo node *v*_*Sj*_ into the community as long as *v*_*Sj*_ is connected with 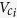, and CLP Conditions (2) and (3) (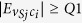) are satisfied. CLP 1 and CLP 2 do not always allow the growth of small communities, as those communities often fail to compete for solo nodes in CLP 1 & 2. This is a CLP which prioritises the growth of the small communities.
- CLP 3/4/5/6 and GNR 3/4/5: In these label propagation processes, a solo node *v*_*Sj*_ has the chance to enter an exempted community provided that the number of edges from the solo node to the exempted communities, 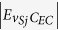, is the highest amongst all the communities that are connected to the solo node, 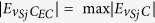. In addition, 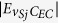 must be 2 times higher than the second highest number of connection from the solo node to the other communities. As mentioned earlier, the constraints on the CLP 3/4/5/6 and GNR 3/4/5 are gradually relaxed in Stage 5. This can be done by modifying CLP Condition (3) for each CLP and GNR. Aside from that, CLP Condition (3) is defined differently depending on whether the *c*_*i*_ is an exempted community or not. Thus, CLP Condition (3) in the CLP 3/4/5/6 and GNR 3/4/5 is defined as follow:

For CLP 3 and GNR 3:
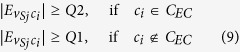


For CLP 4 and GNR 4:
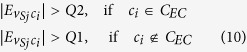


For CLP 5 and GNR 5:
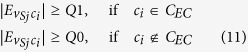
where *Q0, Q1* and *Q2* are the minimum, first quartile and median of the 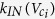.

Finally, the CLP Condition (3) is omitted in CLP 6.

- GM 3: Generally, this process is very similar to GM 2, except that it can handle ties between multiple communities. In case of a tie, the values of RatioB−RatioA for each pair of communities are compared. The pair of communities with the 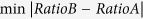 is merged, provided that the modularity does not decrease after the merging. Furthermore, it is a free-for-all GM where all the communities, including the exempted communities, have the chance to merge. Hence, GM Condition (1) is omitted in this process.

### Time Complexity

The time complexity of the initial labelling is represented by 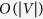. The calculation of the *SDI*, allocation of one degree nodes, and the HTH *SDI* grouping run on 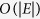. As the solo and grouped nodes are updated separately in the CLP and GNR processes, the time complexity of the propagation process is also split. In general, the time complexity of the CLP and GNR are 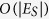 and 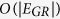, where 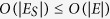, 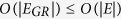 and 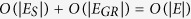. Most of the time, the CLP or GNR process is coupled with the EC process and they are executed until there is a convergence in the labels. Given that the EC process runs in 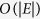 and *t* represents the number of iterations before convergence, the time complexity of the EC-CLP and EC-GNR processes are 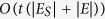 and 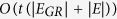, respectively. The group merging process runs in 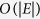 and it is coupled with the EC and GNR processes. Thus, the time complexity of the EC-GM-GNR process is 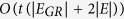.

Stage 4 of the proposed algorithm is iterative. Let *t*_*S*4_ be the number of iterations before Stage 4 reaches a convergence in the labels. The time complexity of Stage 4 is 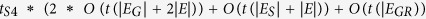. By referring to the algorithm flowchart (see Fig. 3), the time complexity are 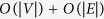, 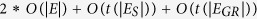, 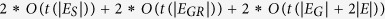 and 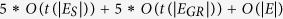 for Stage 1, 2, 3 and 5, respectively.

## Additional Information

**How to cite this article**: Chin, J. H. and Ratnavelu, K. A semi-synchronous label propagation algorithm with constraints for community detection in complex networks. *Sci. Rep.* **7**, 45836; doi: 10.1038/srep45836 (2017).

**Publisher's note:** Springer Nature remains neutral with regard to jurisdictional claims in published maps and institutional affiliations.

## Figures and Tables

**Table 1:** Summary of the generated LFR networks.

Networks	*N*	*C*_*min*_	*C*_*max*_	*μ*
SNSC	1000	10	50	0.1 to 0.8
SNLC	1000	20	100	0.1 to 0.8
LNSC	5000	10	50	0.1 to 0.8
LNLC	5000	20	100	0.1 to 0.8

**Table 2:** Summary of the real-world networks considered in this study.

Networks	Nodes	Edges	Ground Truth
Zachary^32^	34	78	Yes
Dolphins^33^	62	159	Yes
Pol-books^24^	105	441	Yes
Football^34^	115	613	Yes
Jazz^35^	198	2742	No
E. coli^36^	418	519	No
Email^37^	1133	5451	No
Power^21^	4941	6494	No
Pretty Good Privacy^38^	10680	24316	No
arXiv Astro-ph^39^	18771	198050	No
Brightkite^40^	58228	214078	No

**Table 3:** The *NMI* of the real-world networks with ground truth communities.

Networks	SSCLPA	CLPA-GNR	LPA	GANXiS	Infomap	RN	Blondel
Zachary	0.707	**0.837**	0.602	0.707	0.699	0.631	0.687
Dolphins	0.616	0.488	0.645	0.458	0.537	**1.000**	0.645
Pol-books	0.493	0.552	0.554	0.462	0.537	**0.574**	0.569
Football	**0.969**	0.955	0.933	0.951	0.952	**0.969**	0.903

The best detection algorithm for each network is shown in bold.

**Table 4:** The *Q* values of the real-world networks.

Networks	SSCLPA	CLPA-GNR	LPA	GANXiS	Infomap	RN	Blondel
Zachary	0.415	0.303	0.416	0.415	0.402	0.406	**0.420**
Dolphins	0.525	0.513	**0.527**	0.513	0.525	0.379	**0.527**
Pol-books	0.518	0.514	0.526	0.519	**0.527**	**0.527**	**0.527**
Football	0.601	0.579	**0.605**	0.603	0.603	0.601	0.603
Jazz	0.420	0.282	0.443	0.442	0.443	0.288	**0.445**
E. coli	0.735	0.749	0.772	0.757	0.707	0.771	**0.777**
Email	0.525	0.520	0.558	0.540	0.536	0.008	**0.570**
Power	0.884	0.888	0.818	0.797	0.811	0.343	**0.934**
Pretty Good Privacy	0.798	0.852	0.821	0.804	0.857	—	**0.880**
arXiv Astro-ph	0.334	0.294	0.537	0.570	0.581	—	**0.614**
Brightkite	0.538	-	0.640	0.631	0.441	—	**0.664**

The best detection algorithm for each network is shown in bold.

**Table 5:** The *Q*_*ds*_ values of the real-world networks.

Networks	SSCLPA	CLPA-GNR	LPA	GANXiS	Infomap	RN	Blondel
Zachary	0.235	0.182	0.234	0.235	0.217	**0.240**	0.230
Dolphins	0.207	0.187	0.187	**0.216**	0.213	0.136	0.187
Pol-books	0.216	0.183	0.192	**0.225**	0.199	0.190	0.191
Football	**0.491**	0.482	0.449	0.473	0.474	**0.491**	0.417
Jazz	**0.232**	0.187	0.215	0.221	0.220	0.205	0.213
E. coli	0.132	0.116	0.142	**0.154**	0.087	**0.154**	0.116
Email	0.076	0.057	0.050	0.059	**0.088**	0.015	0.041
Power	0.070	0.055	**0.161**	0.149	0.003	0.124	0.019
Pretty Good Privacy	**0.160**	0.064	0.153	**0.160**	0.018	—	0.031
arXiv Astro-ph	0.130	0.097	0.115	**0.145**	0.099	—	0.027
Brightkite	0.037	-	0.027	**0.044**	0.006	—	0.011

The best detection algorithm for each network is shown in bold.

**Table 6:** The computational time of various detection algorithms on the Astro-ph and Brightkite networks.

Networks	SSCLPA	LPA	GANXiS	Infomap	Blondel
Pretty Good Privacy	24 s	7 s	557 s	1 s	0.25 s
arXiv Astro-ph	169 s	83 s	2444 s	5 s	0.22 s
Brightkite	290 s	76 s	1741 s	8 s	0.37 s

The reported time for the LPA is the sum of 100 runs on both networks. On the other hand, the reported time for the GANXiS is the sum of 100 on the Pretty Good Policy and arXiv Astro-ph networks, while 10 runs on the Brightkite networks.
